# Association between dietary factors and colorectal serrated polyps: a systematic review and meta-analysis

**DOI:** 10.3389/fnut.2023.1187539

**Published:** 2023-07-27

**Authors:** Zhixin Zhu, Xifei Guan, Nawen Liu, Xiaoxia Zhu, Sheng Dai, Dehai Xiong, Xiuyang Li

**Affiliations:** ^1^Department of Big Data in Health Science, and Center for Clinical Big Data and Statistics, The Second Affiliated Hospital, College of Medicine, Zhejiang University, Hangzhou, Zhejiang, China; ^2^Department of General Surgery, School of Medicine, Run Run Shaw Hospital, Zhejiang University, Hangzhou, Zhejiang, China; ^3^Department of General Surgery, Three Gorges Hospital, Chongqing University, Chongqing, China

**Keywords:** colorectal serrated polyps, colorectal cancer, dietary factors, meta-analysis, systematic review

## Abstract

**Background:**

Dietary factors may affect the incidence of colorectal serrated polyps (SP). However, its effects on SP are unclear as epidemiological studies on this topic have showed inconsistent results. The present systematic review and meta-analysis sought to evaluate the effects of dietary factors on SPs.

**Methods:**

Studies regarding the association between dietary factors and SPs were identified by searching PubMed, Cochrane library, Embase and Chinese Biomedical Literature database from inception until 27 February 2023. Search terms include serrated, hyperplastic, adenoma, polyps, colorectal, rectal, rectum and risk. Heterogeneity was assessed using *I*^2^ statistics. The meta-analysis was conducted by using a random-effects model, and the pooled effects were expressed with odds ratios (OR) and 95% confidence intervals (95% CI). Probable sources of heterogeneity were identified through meta-regression. Subgroup analysis were based on lesion types, study designs, countries, and so on.

**Results:**

28 studies were ultimately eligible after scanning, and five dietary factors including vitamin D, calcium, folate, fiber and red or processed meat were excerpted. Higher intakes of vitamin D (OR = 0.95, 95%CI:0.90–1.02), calcium (OR = 0.97, 95%CI: 0.91–1.03) and folate (OR = 0.82, 95% CI: 0.6–1.13) were not significantly associated with SP. Fiber intake (OR = 0.90, 95% CI: 0.82–0.99) was a protective factor against SPs. Red meat intake increased the risk of SPs by 30% for the highest versus lowest intakes (OR = 1.30, 95% CI: 1.13–1.51). For different lesion types, higher folate intake was associated with a decreased risk of HPs (OR = 0.59, 95%CI: 0.44–0.79), and higher vitamin D intake decreased the risk of SPs including SSA/P (OR = 0.93, 95%CI: 0.88–0.98).

**Conclusions:**

Higher dietary fiber intake plays an effective role in preventing SP, while red meat intake is associated with an increased risk of SP. This evidence provides guidance for us to prevent SP from a dietary perspective.

**Systematic review registration:**

https://www.crd.york.ac.uk/prospero/display_record.php?, RecordID=340750.

## 1. Introduction

Colorectal cancer (CRC) is considered to be caused by the accumulation of various aberrant mutations in the epithelial cells lining the colorectal mucosa (1). In 2020, there were about 1.932 million new cases of CRC in the world, and the morbidity ranked third among all malignant tumors. There were about 935,000 deaths of colorectal cancer, and the mortality ranked the second among all malignant tumors, causing a serious disease burden to the world (2).

CRC mainly evolves from colorectal adenoma, that is, normal mucosa first appears with epithelial hyperplasia-like changes, and then can gradually transform into an adenoma, which can later develop into carcinoma *in situ* and invasive carcinoma (3, 4). Studies over the past decade have shown that colorectal serrated polyps (SP) also have the potential to progress to CRC, with 10–30% of CRC developing through serrated polyps (5, 6). SPs are most commonly classified as hyperplastic polyps (HP), sessile serrated adenomas/polyps (SSA/P) and traditional serrated adenomas.

The association of dietary factors with colorectal adenomas and CRC has been extensively explored, and the dose-response relationship has also been established. It is reported that the relative risk for developing CRC is 1.38 for a 50 g/day increase in alcohol intake (7), 0.90 for an increase of 10 g/day of dietary fiber (8), 1.24 for 120 a g/day increase of red meat, 1.36 for a 30 g/day increase of processed meat (9). A study showed that a healthy lifestyle can reduce the risk of morbidity and mortality of colorectal adenoma and CRC (10).

Gao et al. conducted a systematic review and meta-analysis, revealing a strong positive relationship between proximal SP and synchronous advanced neoplasia (11). However, HPs, the most prevalent type of SP, have a relatively lower chance to become cancerous (12). Many investigators have also investigated the relationship between different dietary factors and CRC precursor lesions, but the results remain controversial (13–18). The aim of this systematic review and meta-analysis, therefore, is to evaluate the association between dietary factors, including vitamin D, calcium, folate, fiber, and red meat, with the risk of SP.

## 2. Materials and methods

### 2.1. Search strategy

This meta-analysis was registered on PROSPERO (No. CRD42022340750) (19) and compliant with the main PRISMA statement (20, 21). Randomized controlled trials (RCT), cohort studies, case-control studies and cross-sectional studies published before 27 February 2023 were collected from electronic databases such as PubMed, Cochrane Library, Embase and China BioMedical Literature database.

The search terms were: (Serrated OR Hyperplastic) AND (Adenoma OR Polyps) AND (Colorectal OR Rectal OR Rectum) AND Risk (Supplementary material). Besides, the references of all the retrieved articles were checked to identify further relevant articles.

### 2.2. Selection criteria

The following inclusion characteristics based on “PICO(S)” criteria were agreed for screening papers: (1) Adults aged 18 years and over, undergoing endoscopic investigation of the colorectum. (2) Assessment of a dietary factor, for example, vitamin D, calcium, folate, fiber and red meat. (3) Comparisons of the risk of SPs between dietary factors exposure group and non-exposure (or lower exposure) group. (4) Risk of serrated colorectal polyps, encompassing HP, SSA/P and/or TSA. (5) Original studies in English or Chinese.

The exclusion criteria were as follows: (1) Study populations of other co-morbidities, for example, Crohn's Disease, ulcerative colitis, Barrett's esophagus, acromegaly; (2) Studies did not provide sufficient data for a meta-analysis; (3) The control group comprised a population with serrated polyps. (4) Reviews, comments, letters, and animal studies.

### 2.3. Data extraction and quality assessment

Two reviewers (ZX Zhu and XF Guan) used Endnote X9 software to screen titles and abstracts independently. All data were extracted by two researchers (NW Liu and XX Zhu) independently using a standardized form. If there was any disagreement between two reviewers, the report would be sent to a third researcher (XY Li) and fully discussed. Data extraction included: the name of the first author, published year, country, study design, age, cases, sample size, type of SPs, dietary factors and adjusted confounders. The Newcastle-Ottawa scale was used to assess the quality and bias risk of observational studies, and a study with 7 scores (or more) was defined as a high-quality study. The Cochrane collaboration tool were used to assess the quality of RCTs (22).

### 2.4. Statistical analysis

If studies reported results from the same population and their investigated factors overlapped, the most recent publications or studies with the biggest sample size were used for meta-analysis. Pooled ORs of dietary factors with 95 % CI for the risk of SPs were calculated using random effects models for the highest v. the lowest categories. The between-study heterogeneity was assessed by Cochran's *Q*-test and *I*^2^ statistic, and heterogeneity was considered to be significant if *I*^2^ > 50% and *P* < 0.05. The summary OR was calculated through the random-effects model, which takes into account the presence of heterogeneity in their calculations (23). Forest plots were drawn to show each study's result and estimate the pooled effect sizes. A sensitivity analysis was performed to evaluate the stability of the results. Each time one study was omitted to evaluate the risk estimate. Subgroup analyses were further conducted according to lesion type (HP or SP/SSA/P), study design (cross-sectional, case-control, cohort or RCT), country (USA or others) and adjustments including smoking, alcohol intake or body mass index (BMI), to explore the potential source of heterogeneity, if data were permitted. Besides, we conducted sensitivity analyses to assess the stability, and reliability of the pooled estimates by omitting one estimate at a time sequentially and recalculating the pooled results. The stability and reliability were confirmed if no single study altered the significance of the pooled estimate. Furthermore, publication bias was assessed through funnel plot and Egger's linear regression test (24). All statistical analysis was performed with STATA software (Version 17.0; Stata Corporation, College Station, TX, USA). *P* ≤ 0.05 were considered statistically significant.

## 3. Results

### 3.1. Search results and study characteristics

**Figure 1:**
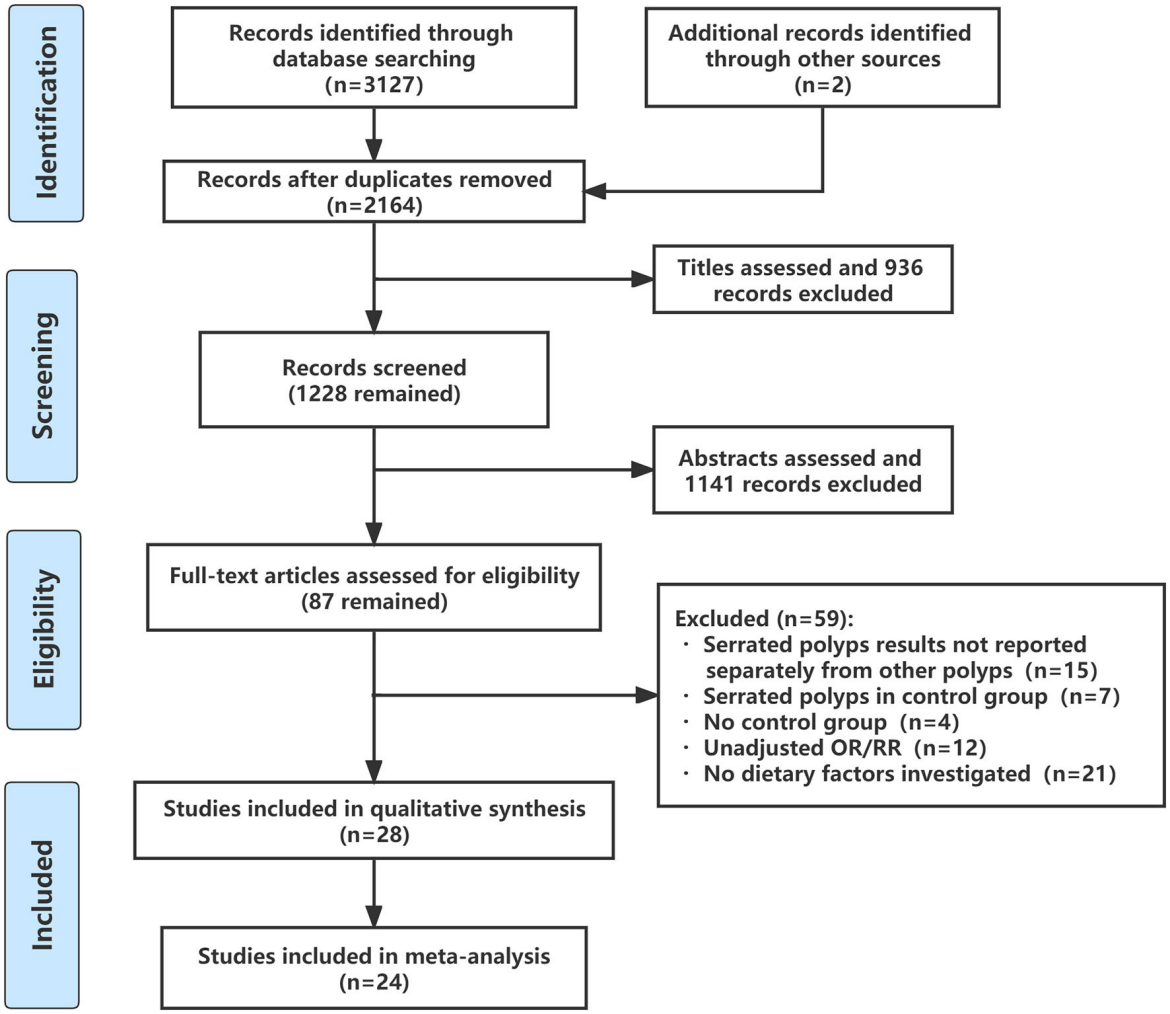
Flow diagram of study selection.

The flow through the selection process was described in a modified PRISMA diagram (Figure 1). A total of 3,129 articles were retrieved from the databases and reference lists during the initial screening process. After a full-text review, 28 studies were included for qualitative analysis, among which four studies reported the same population (13, 14, 16, 25), so 24 studies were left for meta-analysis (15, 17, 18, 26–46). Table 1 summarized the basic characteristics of each study. There were six cross-sectional studies, 12 case-control studies, five cohort studies, and five RCTs. Assessments for risk bias of included observational studies and RCTs were shown in Supplementary Table 1, Supplementary Figure 1, respectively. According to the Newcastle-Ottawa scale and Cochrane collaboration tool, 18 observational studies were rated as high-quality studies. The included RCTs were not at appreciable risk of bias.

**Table 1:** Characteristics of included studies.

**References Location**	**Age^†^**	Study design ^*^	**Cases**	**Control/ sample size**	**Assessment method of factors**	**Factors/ Comparison (highest v. lowest)**	**Outcome**	**Results**	**Adjusted confounders**
Kearney et al. (14) USA	30–55	Prospective cohort (NHS)	175	15,984	Two 61-item and 121-item FFQs	Vit D; Folate (≥ 569 v. < 198 g/d); Fiber (≥ 22 v. < 12.1 g/d)	Distal HP	Vit D: aRR, 0.99 (95%CI, 0.65–1.51); Folate: aRR, 0.45 (95%CI, 0.28- 0.74); Fiber: aRR, 0.79 95%CI, 0.48–1.35	Age, sex#, smoking, alcohol intake, energy intake, family history, previous endoscopy.
Kearney et al. (14) USA	40–75	Prospective cohort (HPFS)	219	12,922	131-item FFQ	Vit D; Folate (≥672 v. < 280 μg/d); Fiber (≥29.3 v. < 17.1 g/d)		Vit D: aRR, 0.99 (95%CI, 0.65- 1.51); Folate: aRR, 0.74 (95%CI, 0.49–1.11); Fiber: aRR, 0.96 (95%CI, 0.46–1.25)	
Martinez et al. (26) Spain	57.3 (9.7)	Cross-sectional study	81	480	Interviewed 138-item FFQ	Calcium (≥1,094 v. ≤ 558 mg/d); Fiber (≥ 28 v. ≤ 15.8 g/d)	HP	Calcium: aOR, 0.32 (95%CI, 0.11–0.96); Fiber: aOR, 0.3 (95%CI, 0.1–0.88)	Age, sex, race, BMI, smoking, energy intake, NSAIDs use.
Platz et al. (16) 1997 USA	40–75	Prospective cohort (HPFS)	327	16,448	131-item FFQ	Fiber (median of Q5 v. median of Q1: 32.3 v. 11.6 g/d)	HP	Fiber: aRR, 0.82 (95%CI, 0.5–1.36)	Age, sex#, BMI, family history, smoking, multivitamin use, physical activity, aspirin use, intake of alcohol, energy, red meat, folate, and methionine, endoscopy prior to 1986.
Erhardt et al. (27) Germany	56.5	Hospital-based case-control	71	224	Interviewed diet history	Red or processed meat (> 15 v. ≤ 15 g/d)	HP	Red or processed meat: aOR, 3.24 (95%CI, 1.23– 140.8)	Age, sex, BMI, smoking, alcohol intake
Morimoto et al. (28) USA	30–74	Hospital-based case-control	219	708	FFQ	Vit D (≥ 461 v. < 135 IU/d); Calcium (≥1,276 v. < 600 mg/d)	HP	Vit D: aOR, 1.6 (95%CI, 0.8–2.9); Calcium: aOR, 0.76 (95%CI, 0.55–1.06)	Age, sex, smoking, alcohol intake, BMI, HRT
Lieberman et al. (29) USA	50–75	Hospital-based case-control	391	1441	FFQ	Vit D (continuous in 100–IU increments); Calcium (continuous in 100–IU increments); Fiber (continuous in 1–g increments)	HP	Vit D: aOR, 1 (95%CI, 0.99–1.01); Calcium: aOR, 1 (95%CI, 0.99–1.01); Fiber: aOR, 1 (95%CI, 0.98- 1.01)	Age
Wallace et al. (30) USA	61 (9.1)	RCT (Calcium Polyp Prevention Study)	279	913	Supplement	Calcium (1,200 mg/day v. placebo)	HP	Calcium: aRR, 0.82 (95%CI, 0.67–1.00)	Age, sex, clinical center
Dai et al. (25) USA	57.7	Hospital-based case-control (Tennessee Colorectal Polyp Study)	210	1306	Telephone survey, FFQ	Calcium (< 1129 v. > 687 mg/d)	HP	Calcium: aOR, 1.01 (95%CI, 0.57–1.79)	Age, sex, race, BMI, smoking, alcohol intake, education, physical activity, recruitment site, intakes of total energy intake, saturated fat, folate, vitamin E, retinol equivalent, zinc, vitamin B-6, fiber,vitamin D, and magnesium.
Wallace et al. (17) USA	57.5 (9.6)	Pooled data from RCTs and nested case-control studies within these	812	2018	FFQ	Fiber (Q4 v. Q1); Red or processed meat (Q4 v. Q1)	SP	Left colon: Fiber: aRR, 0.88 (95%CI, 0.72–1.08); Red or processed meat: aOR, 1.17 (95%CI, 0.93–1.48) Right colon: Fiber: aOR, 0.95 (95%CI, 0.67–1.36); Red or processed meat: aOR, 1.03 (95%CI, 0.68–1.57)	Age, sex, smoking, log calories, study center
Adams et al. (31) USA	NR	Cross-sectional study	85	225	LC MS (25-hydroxy-vitamin D)	25-hydroxy-vitamin D (> 28.9 v. ≤ 20.5 ng/ml)	HP	Vit D: aOR, 1.17 (95%CI, 0.55–2.51)	Age, sex, BMI, smoking, supplement use, physical activity, previous polyp diagnosis, season of blood draw,
Burnett-Hartman et al. (32) USA	20–74	Hospital-based case-control	691	772	Telephone interview	Red or processed meat (> 3 v. 0 servings/wk)	HP	Red or processed meat: aOR, 1.34 (95%CI, 0.92–1.94)	Age, sex, race, education, BMI, smoking, alcohol intake, NSAIDs use, HRT.
Fu et al. (13) USA	56.7 (7.0)	Hospital-based case-control (Tennessee Colorectal Polyp Study)	662	3,764	Telephone survey, FFQ	Calcium (≥1,169.3 v. ≤ 829.3 mg/d); Folate (≥584.4 v. ≤ 421.4 μg/day); Fiber ( ≤ 24.1 v. ≥16.6 g/d); Red or processed meat (≥ 44.2 v. ≤ 10 g/d)	HP	Calcium: aOR, 0.73 (95%CI, 0.56–0.96); Folate: aOR, 0.73 (95%CI, 0.55–0.96); Fiber: aOR, 0.84 (95%CI, 0.64–1.1); Red or processed meat: aOR, 1.14 (95%CI, 0.93–1.39)	Age, sex, race, education, BMI, smoking, NSAID use, total energy intake, study sites, indication for colonoscopy, recruitment before or after colonoscopy, year of recruitment.
Crockett et al. (33) USA	NR	Pooled data from cross-sectional studies	39	1,316	FFQ	Fiber; Red or processed meat	SSA	Fiber: aOR, 0.73 (95%CI, 0.5–1.07); Red or processed meat: aOR, 1.18 (95%CI, 0.81–1.71)	Age, sex
Shuai et al. (34) China	59	Hospital-based case-control	30	258	Enzyme-linked immunosorbent assay (plasma 25-hydroxy-vitamin D)	25-hydroxy-vitamin D (≥ 18.23 v. < 13.39 ng/mL)	HP	Vit D: aOR, 0.25 (95%CI, 0.08–0.77)	Age
Rees et al. (35) USA/Canada	21–80	RCT (Aspirin/Folate Polyp Prevention Study)	167	643	Supplement/LC MS (plasma methylated folate)	Methylated folate (37.35 < v. > 85.5 nmol/L)	SP	Folate: aRR, 0.61 (95%CI, 0.36–1.23)	Age, sex
Davenport et al. (36) USA	57.2 (7.7)	Hospital-based case-control (Tennessee Colorectal Polyp Study)	774	3,851	Telephone survey, FFQ	Calcium (>1,217 v. < 595.8 mg/d); Folate (>811.8 v. < 394.7 ug/d); Fiber (≥ 24.73 v. < 12.88 g/d); Red or processed meat (> 73.38 v. 16.06 g/d)	HP; SSA/P	HP: Calcium: aOR, 0.86 (95%CI, 0.55–1.35); Folate: aOR, 0.57 (95%CI, 0.34–0.95); Fiber: aOR, 1.09 (95%CI, 0.68–1.76); Red or processed meat: aOR, 1.48 (95%CI, 1.03–2.14) SSP:Calcium: aOR, 0.7 (95%CI, 0.33–1.45); Folate: aOR, 1 (95%CI, 0.44–2.29); Fiber: aOR, 0.47 (95%CI, 0.22–1.01); Red or processed meat: aOR, 2.59 (95%CI, 1.41–4.74)	Age, sex, BMI, smoking, educational attainment, year of colonoscopy, study site, total daily energy intake, NSAID use, fat intake.
He et al. (37) USA	60.2 (10.6)	Prospective cohort (NHS, NHS II, and HPFS)	7,945	141,143	FFQ	Vit D (Q4 v. Q1); Calcium (Q4 v. Q1); Folate (Q4 v. Q1); Fiber: (Q4 v. Q1); Red or processed meat (Q4 v. Q1)	SP	Vit D: aOR, 0.92 (95%CI, 0.86–0.98); Calcium: aOR, 1 (95%CI, 0.93–1.07); Folate: aOR, 1.04 (95%CI, 0.97–1.11); Fiber: aOR, 0.97 (95%CI, 0.9–1.04); Red or processed meat: aOR, 1.06 (95%CI, 0.99–1.13)	Age, sex, race, BMI, smoking, physical activity, alcohol intake, aspirin use, family history, cohort, time period of endoscopy, number of prior endoscopies, time in years since the most recent endoscopy, reason for endoscopy.
Passarelli et al. (41) USA/Canada	57 (10)	RCT (Aspirin/Folate Polyp Prevention Study)	24	490	Supplement	Folate (1 mg/d v. placebo)	SSA/P	Folate: aRR, 1.38 (95%CI, 0.59–3.19)	Age, sex, center, race, BMI, smoking, family history
Crockett et al. (38) USA	58.1 (6.8)	RCT (Vitamin D/Calcium Polyp Prevention Study)	565	2,058	Supplemental VD and calcium	Vit D (1,000 IU/d v. placebo); Calcium (1,200 mg/d v. placebo)	SP	Vit D: aRR, 1.01 (95%CI, 0.87–1.17); Calcium: aRR, 1.15 (95%CI, 0.98–1.36)	Age, sex, race, BMI, smoking, clinical center, anticipated, surveillance interval, randomization arm of randomization, number of baseline serrated polyps.
Gurjar et al. (39) USA	NR	Pooled data from cross-sectional studies	81	1,482	FFQ	Calcium; Folate; Fiber; Red or processed meat	SSA/P	Calcium: aOR, 1.17 (95%CI, 0.58–2.36); Folate: aOR, 0.57 (95%CI, 0.29–1.11); Fiber: aOR, 1.99 (95%CI, 0.94–4.21); Red or processed meat: aOR, 1.66 (95%CI, 0.87–3.17)	Age, sex, total energy intake, year of study
Ivancovsky-Wajcman et al. (40) Israel	40–70	Case-control study	NR	386	FFQ	Red or processed meat (>0.33 v. < 0.33 portions/week)	Distal HP	Red or processed meat: aOR, 2.04 (95%CI, 1.02–4.05)	Not report
Mosley et al. (15) USA	57.2	Hospital-based case-control (Tennessee Colorectal Polyp Study)	212	3,803	Telephone survey, FFQ	Red or processed meat (≥ 75.7 v. < 16.3 g/d)	SSA/P	Red or processed meat: aOR, 2.38 (95%CI, 1.44–3.93)	Age, sex, race, BMI, study site, alcohol intake, smoking, physical activity, total energy intake, NSAIDs use, educational attainment, indication for colonoscopy.
Song et al. (43) USA	67.1 (7.1)	RCT (VITamin D and OmegA-3 TriaL)	341	25,871	Supplement	Vit D (2,000 IU/d v. placebo)	SP	Vit D: aOR, 1.02 (95%CI, 0.82–1.26)	Age, sex, and fish oil treatment assignment, use of colonoscopy
Yoo et al. (18) South Korea	53.3 (9.5)	Cross-sectional study	4,864	31,004	Supplement/LC MS (serum 25-hydroxy-vitamin D)	25-hydroxy-vitamin D (≥ 30 v. < 20 ng/mL)	HP	Vit D: aOR, 0.91 (95%CI, 0.81–1.02)	Age, sex, BMI, smoking, alcohol drinking, physical activity, family history, NSAIDs use
Kim et al. (42) USA	< 50y	Prospective cohort (NHS II)	1,878	29,186	FFQ	Vit D (≥ 600 v. < 300 IU/d)	SP	Vit D: aOR, 0.85 (95%CI, 0.7–1.03)	Age, sex#, race, BMI, alcohol intake, smoking, energy intake, red and processed meat intake, fiber intake, folate intake, NSAIDs ues, physical activity, TV viewing time, family history, time period of endoscopy, time since most recent endoscopy, number of reported endoscopies, and reason for current endoscopy.
Hang et al. (46) USA	60.3 (10.6)	Prospective cohort (NHS, NHS II, and HPFS)	10,478	142,052	FFQ	Red or processed meat (median of Q5 v. median of Q1: 0.49 v. 0.04 servings/d)	SP	Red or processed meat: aOR, 1.13 (95%CI, 1.06–1.21)	Age, sex, race, cohort (NHS, NHS II, or HPFS), time period of endoscopy, number of prior endoscopies, the most recent endoscopy time, family history, alcohol intake, physical activity, smoking, aspirin use, menopausal status, postmenopausal hormone use.
O'Sullivan et al. (45) Canada	58.7 (7.2)	Cross-sectional study	247	1,384	Canadian Diet History Questionnaire	Vit D (≥ 600 v. < 600 IU/d); Calcium (≥1,200 v. < 1,200 IU/d); Fiber (≥ 20 v. < 20 g/d)	SP	Vit D: aRR, 0.9 (95%CI, 0.71–1.14); Calcium: aRR, 0.79 (95%CI, 0.62–1); Fiber: aRR, 0.8 (95%CI, 0.62- 1.03)	Age, sex#, race, BMI, smoking, alcogol intake, NSAID use, family history, reason for colonoscopy, time period of endoscopy
Anthony et al. (44) Australia	29–55	Hospital-based case-control	350	714	Self-reported questionnaire	Calcium (one dose increase per week); Fiber (one dose increase per week)	SP	Calcium: aOR, 0.97 (95%CI, 0.89–1.05); Fiber: aOR, 0.82 (95%CI, 0.75- 0.9)	Age, sex, BMI, smoking, alcogol intake, aspirin use, NSAIDs, use, multivitamins intake, HRT, pregnancy

† The age variable was described in terms of mean (standard deviation) or age range.

* If studies reported results from the same population and their investigated factors overlapped, the most recent publications or studies with the biggest sample size were used for meta-analysis. #Homogenous study population (men or women).

NHS, Nurses Health Study; HPFS, Health Professionals Follow-Up Study; RCT, randomized controlled trial; NR, not report; FFQ, food frequency questionnaire; LC MS, liquid chromatography tandem mass spectrometry; HP, hyperplastic polyp; SSP/A, sessile serrated polyp/adenoma; SP, serrated polyp; Vit D, vitamin D; BMI, body mass index; NSAID, non-steroidal anti-inflammatory drug; HRT, hormone replacement therapy.

### 3.2. Dietary factors and risk of colorectal serrated polyps

**Figure 2:**
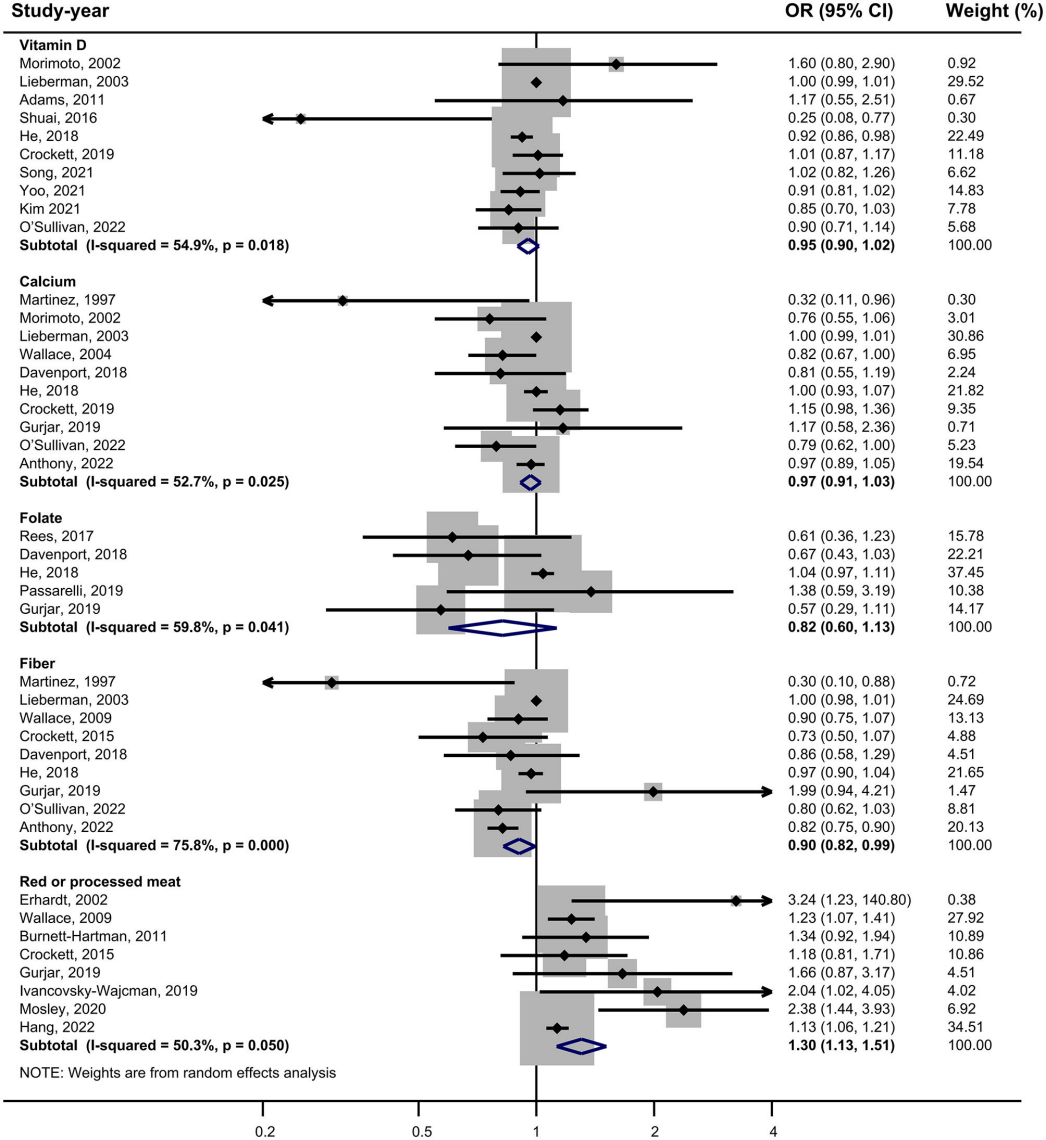
Forest plot of highest v. lowest category of dietary factors and serrated polyp risk.

Vitamin D: 10 studies investigated the association between vitamin D and the risk of SPs (18, 28, 29, 31, 34, 37, 38, 42, 43, 45). The forest plot illustrated a close-to-significant decreased risk of SPs with increased levels of vitamin D intake (OR = 0.95, 95%CI:0.90–1.02), and statistically significant heterogeneity was found (*I*^2^ = 54.9%; *P* = 0.018) (Figure 2). Subgroup analysis showed that vitamin D may be related to a statistically significant decrease in SP risk in cohort studies (OR = 0.91, 0.86–0.97) (Table 2). Besides, similar associations were observed when studies adjusted for smoking (OR = 0.93, 95%CI:0.88–0.97), alcohol intake (OR = 0.91, 95%CI: 0.87–0.96) or BMI (OR = 0.93, 95%CI:0.88–0.97). Also, these adjustments were identified as sources of heterogeneity by meta-regression analyses.

**Table 2:** Subgroup analysis of vitamin D, calcium, folate, fiber and red or processed meat and gastric cancer risk.

**Subgroup**	**Number**	**OR (95% CI)**	**Heterogeneity**		
			**I**^2^ **(%)**	***P*** **heterogeneity**	***P*** between subgroups^*^
**Vitamin D (overall effect)**	10	0.95 (0.90, 1.02)	54.9	0.018	
Country					0.161
USA	7	0.97 (0.92, 1.03)	45.8	0.086	
Others	3	0.86 (0.67, 1.10)	59.6	0.084	
Study design					0.763
Cross-sectional	3	0.91 (0.82, 1.01)	0.0	0.808	
Case-control	3	0.89 (0.46, 1.71)	74.4	0.020	
Cohort	2	**0.91 (0.86, 0.97)**	0.0	0.447	
RCT	2	1.01 (0.90, 1.15)	0.0	0.941	
Adjusted for smoking					**0.037**
Yes	7	**0.93 (0.88, 0.97)**	0.0	0.495	
NO	3	0.96 (0.77, 1.20)	65.5	0.055	
Adjusted for alcohol intake					**0.018**
Yes	4	**0.91 (0.87, 0.96)**	0.0	0.477	
NO	5	1.00 (0.91, 1.10)	33.0	0.201	
Adjusted for BMI					**0.037**
Yes	7	**0.93 (0.88, 0.97)**	0.0	0.495	
NO	3	0.96 (0.77, 1.20)	65.5	0.055	
**Calcium (overall effect)**	10	0.97 (0.91, 1.03)	52.7	0.025	
Country					0.472
USA	7	0.99 (0.93, 1.05)	43.3	0.102	
Others	3	0.84 (0.64, 1.10)	68.6	0.041	
Study design					0.342
Cross-sectional	3	0.77 (0.47, 1.25)	48.4	0.144	
Case-control	4	0.98 (0.93, 1.04)	30.7	0.228	
Cohort	1	1.00 (0.93, 1.07)	-	-	
RCT	2	0.98 (0.70, 1.36)	84.8	0.010	
Adjusted for smoking					0.958
Yes	7	0.95 (0.86, 1.05)	58.6	0.024	
NO	3	0.95 (0.81, 1.10)	49.5	0.138	
Adjusted for alcohol intake					0.679
Yes	4	0.95 (0.87, 1.03)	47.2%	0.128	
NO	6	0.96 (0.84, 1.10)	58.9%	0.033	
Adjusted for BMI					0.958
Yes	7	0.95 (0.86, 1.05)	58.6	0.024	
NO	3	0.95 (0.81, 1.10)	49.5	0.138	
**Folate (overall effect)**	5	0.82 (0.60, 1.13)	59.8	0.041	
Study design					0.380
Cross-sectional	1	0.57 (0.29, 1.12)	-	-	
Case-control	1	0.67 (0.43, 1.04)	-	-	
Cohort	1	1.04 (0.97, 1.11)	-	-	
RCT	2	0.87 (0.39, 1.92)	57.4	0.125	
Adjusted for smoking					0.198
Yes	3	0.95 (0.68, 1.31)	53.1	0.119	
NO	2	**0.59 (0.38, 0.93)**	0.0	0.884	
Adjusted for alcohol intake					0.081
Yes	1	1.04 (0.97, 1.11)	-	-	
NO	4	**0.69 (0.52, 0.94)**	2.4	0.380	
Adjusted for BMI					0.198
Yes	3	0.95 (0.68, 1.31)	53.1	0.119	
NO	2	0.59 (0.38, 0.93)	59.8	0.041	
**Fiber (overall effect)**	9	**0.90 (0.82, 0.99)**	75.8	< 0.001	
Country					**0.013**
USA	6	0.97 (0.91, 1.04)	40.1	0.138	
Others	3	**0.79 (0.66, 0.95)**	39.0	0.194	
Study design					0.380
Cross-sectional	4	0.82 (0.53, 1.27)	66.7	0.029	
Case-control	4	0.90 (0.79, 1.04)	86.6	0.000	
Cohort	1	0.97 (0.90, 1.04)	-	-	
Adjusted for smoking					0.330
Yes	6	**0.87 (0.78, 0.97)**	61.3	0.024	
NO	3	1.00 (0.71, 1.41)	65.9	0.053	
Adjusted for alcohol intake					0.619
Yes	3	0.88 (0.76, 1.01)	77.6	0.011	
NO	6	0.92 (0.77, 1.09)	59.8	0.029	
Adjusted for BMI					0.401
Yes	5	**0.86 (0.74, 0.99)**	69.1	0.012	
NO	4	0.96 (0.81, 1.12)	58.3	0.066	
**Red or processed meat (overall effect)**	8	**1.30 (1.13, 1.51)**	50.3	0.050	
Country					0.233
USA	6	**1.27 (1.10, 1.46)**	54.1	0.054	
Others	2	**2.11 (1.09, 4.10)**	0.0	0.713	
Study design					0.520
Cross-sectional	5	1.29 (0.93, 1.78)	0.0	0.370	
Case-control	2	**1.55 (1.15, 2.07)**	52.3	0.078	
Cohort	1	**1.13 (1.06, 1.21)**	-	-	
Adjusted for smoking					0.694
Yes	5	**1.28 (1.08, 1.51)**	61.7	0.034	
NO	3	**1.42 (1.03, 1.95)**	9.8	0.330	
Adjusted for alcohol intake					0.874
Yes	4	**1.46 (1.01, 2.11)**	69.1	0.021	
NO	4	**1.26 (1.11, 1.43)**	0.0	0.422	
Adjusted for BMI					0.110
Yes	3	**1.77 (1.10, 2.86)**	43.7	0.169	
NO	5	**1.18 (1.08, 1.30)**	20.8	0.282	

* The between-subgroup heterogeneity was assessed using univariate meta-regression analysis. Bold indicates statistical significance.

RCT, randomized controlled trial.

Calcium: based on 10 studies (26, 28–30, 36–39, 44, 45), the pooled OR (95% CI) for higher intake of calcium compared to lower intake was 0.97 (0.91–1.03) (Figure 2), and moderate heterogeneity was found (*I*^2^ = 52.7%; *P* = 0.025). In all subgroups, calcium intake was not associated with risk of SPs, and countries, study designs and adjustments did not influence the heterogeneity of included studies (Table 2).

Folate: for folate, five studies were included in the pooled analysis (35–37, 39, 41), which indicated that folate intake was not statistically associated with SPs (OR = 0.82, 95% CI: 0.60–1.13) (Figure 2), and we identified moderate heterogeneity (*I* ^2^ = 59.8%; *P* = 0.041). Protective effects were detected in studies not adjusting smoking (OR = 0.59, 95% CI:0.38–0.93) or alcohol intake (OR=0.69, 95% CI:0.52–0.94) (Table 2).

Fiber: Nine studies investigating the risk of SPs with regard to the level of fiber intake were involved in the meta-analysis (17, 26, 29, 33, 36, 37, 39, 44, 45). The result showed that the risk of SPs in the highest fiber intake group decreased by 10% compared with the lowest fiber intake group (OR = 0.90, 95% CI: 0.82–0.99) (Figure 2), and large heterogeneity was discovered (*I*^2^= 75.8%; *P* < 0.001). Meta-regression showed statistically significant differences in the heterogeneity of subgroups with different countries (Table 2). Studies conducted in the USA did not reveal a statistical correlation between fiber intake and SP risk (OR = 0.97, 95% CI:0.91–1.04), while studies in other counties found fiber intake had a protective effect against SPs (OR = 0.79, 95% CI:0.66–0.95).

Red or processed meat: the pooled result of eight studies (15, 17, 27, 32, 33, 39, 40, 46) showed that higher red or processed meat intake was associated with an increased risk of SP (OR = 1.30, 95% CI: 1.13–1.51), and moderate heterogeneity was observed (*I*^2^= 50.3%; *P* = 0.050). This association was found in almost all subgroups (Table 2).

**Figure 3:**
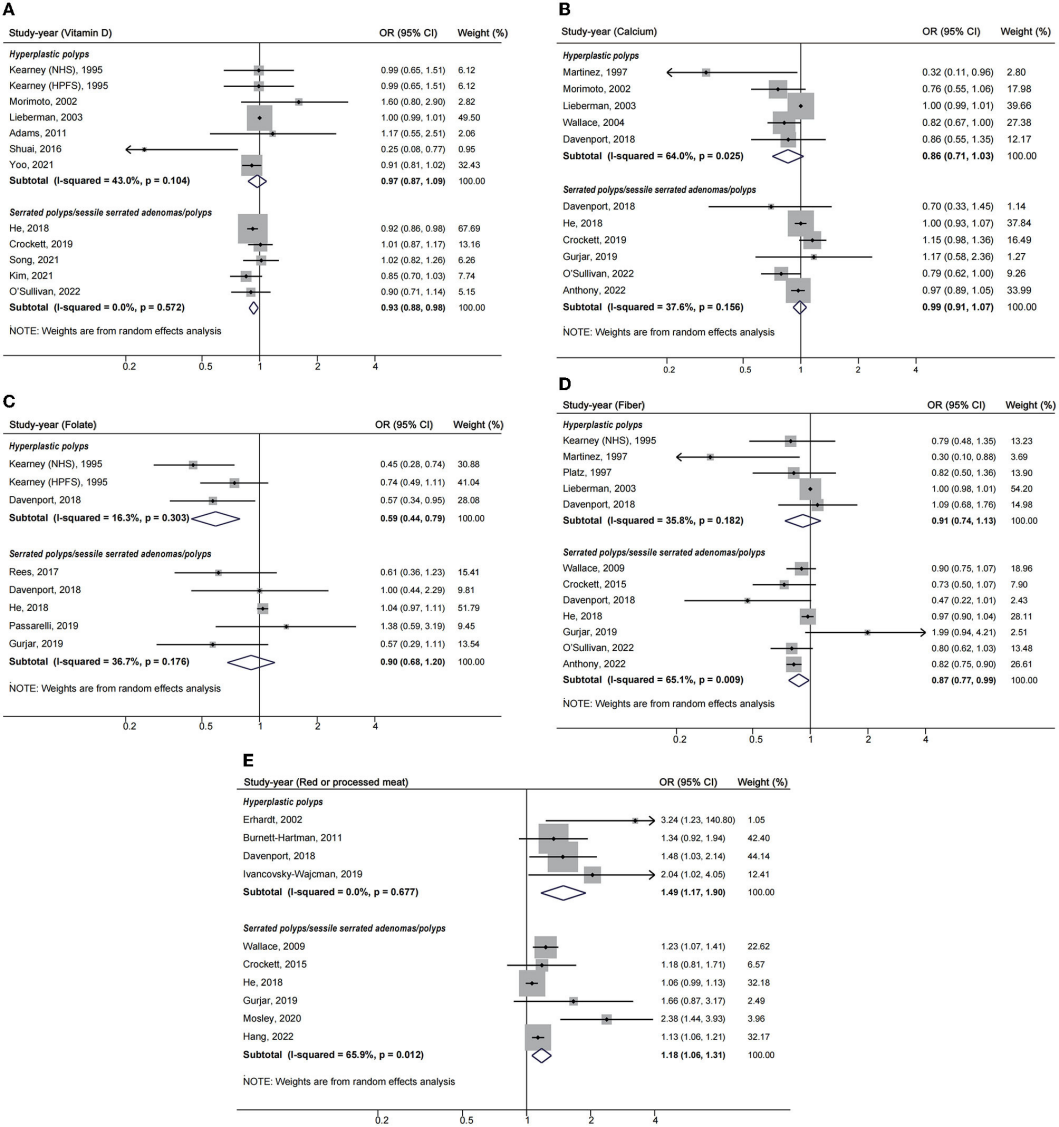
Forest plots of highest v. lowest category of dietary factors and serrated polyp risk for different lesion types. **(A)** Vitamin D; **(B)** calcium; **(C)** folate; **(D)** fiber; **(E)** red or processed meat.

For different lesion types, as was shown in Figure 3, higher folate intake was associated with a decreased risk of HP (OR = 0.59, 95%CI: 0.44–0.79), and higher vitamin D intake decreased the risk of SP including SSA/P (OR = 0.93, 95%CI: 0.88–0.98). Fiber was perhaps a protective factor for SP and SSA/P (OR = 0.87, 95%CI: 0.77, 0.99), but not for HP (OR = 0.91, 95%CI: 0.74–1.13).

### 3.3. Publication bias and sensitivity analysis

**Figure 4:**
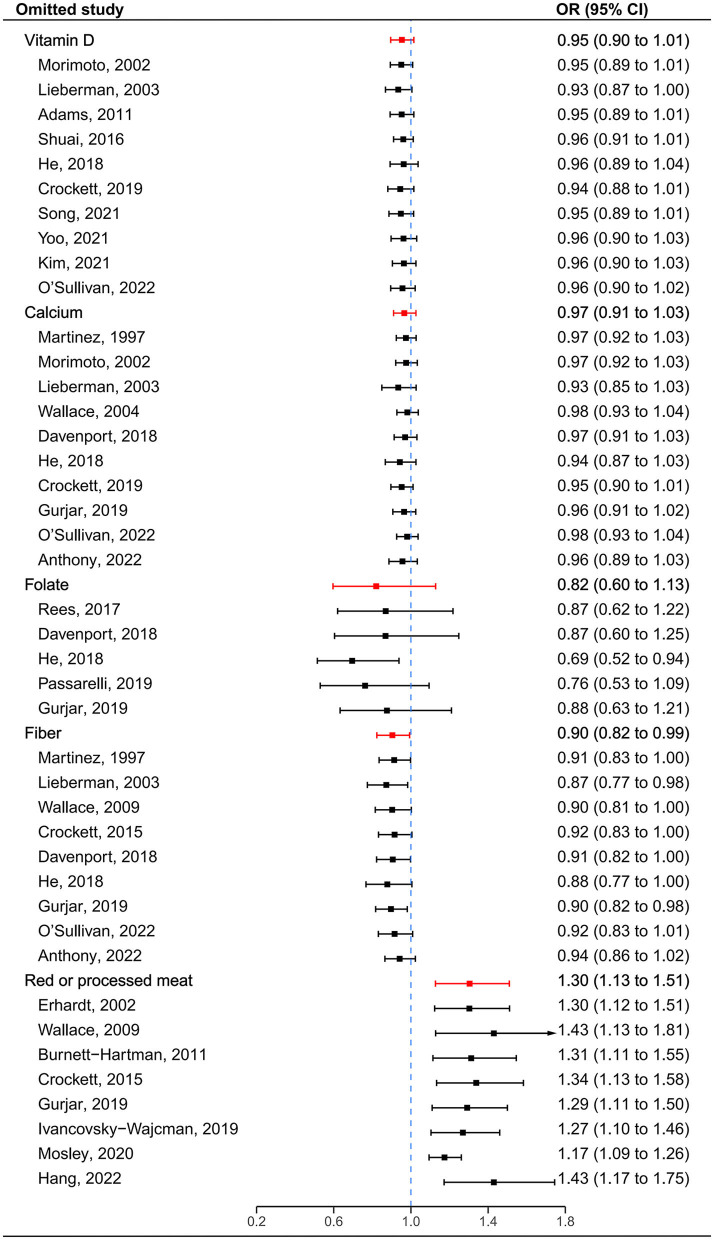
Sensitivity analyses by omitting one study at a time. The red lines represent the confidence intervals when all estimates are pooled for each dietary factor. The blue dashed line represents the null.

After visual inspection, we found asymmetries in funnel plots (Supplementary Figure 2). However, Egger's regression tests showed that a significant publication bias was indicated only for red or processed meat (*t* = 3.67, *P* = 0.010) (Supplementary Table 2). Following sensitivity analysis, the exclusion of any single study did not affect overall estimates for the influence of vitamin D, calcium and red or processed meat on SP risk, but the significance of pooled estimates of folate and fiber was changed when some studies were removed (Figure 4).

## 4. Discussion

This systematic review and meta-analysis is the latest and most comprehensive study investigating dietary factors and their influence on risk of serrated colorectal polyps. Meta-analyses revealed a statistically significant increased risk of SP associated with red or processed meat intake, and fiber intake was protective against SP. Higher intakes of vitamin D, calcium and folate were not significantly associated with SP risk.

Together, vitamin D and calcium are involved in regulating inflammation, differentiation, apoptosis and carcinogenesis in various cells, and have been widely studied as protective factors against colorectal cancer (47). Lopez-Caleya et al. conducted a meta-analysis based on case-control studies, showing that dietary intake of vitamin D can reduce the risk of CRC by 4% (48). Another more comprehensive meta-analysis done by Huang et al. showed that vitamin D intake and calcium intake were protective factors for colorectal adenomas, and a significant dose-response was observed between the intake of vitamin D or calcium and colorectal tumor incidence (49). According to our results, though vitamin D intake did not reduce the risk of HP, it significantly reduced risk of SP including SSA/P by 7%. Besides, inverse associations between vitamin D intake and SP risk were also observed when studies adjusted for smoking, alcohol intake or BMI. Therefore, we assumed that vitamin D had a certain protective effect on SPs, but more high-quality studies were still needed to confirm this conclusion. Calcium intake did not play a protective role against SP. In an RCT, Crockett et al. revealed that supplementary calcium and the combination of calcium and vitamin D3 even increased the risk of SSA/Ps (38).

Folate is abundant in leafy green vegetables, legumes, and cereals. It has been a focus of CRC chemo-prevention research for several decades because of its role in DNA methylation, repair, and nucleotide synthesis (50). In a randomized controlled trial and a cohort study, both folate supplementation and dietary folate are beneficial for the primary prevention of colorectal adenomas (51, 52). Moazzen et al. conducted a meta-analysis about folate and CRC risk, the results showed higher folic acid intake significantly reduced CRC risk by 23% and 29% in case-control studies and cohort studies, respectively, whereas folic acid supplementation had no significant effect (53). However, in the meta-analysis, neither observational studies nor RCTs suggested folate intake decreased the risk of SP (41). Ree et al. investigated the concentration of serum folates and risk of SPs, finding that people with methylated serum folate concentrations between 37.35 and 60.44 nmol/L had lower SP risk compared with those with concentrations lower than 37.35 nmol/L, but much higher serum folate concentration produced non-significant effect statistically, which might indicate a U-shaped dose-response relationship between folate and SP risk and provided more direct evidence (35). Overall, differences in subject baseline characteristics, methods of folate status assessment, and the biologically effective dose of folic acid in the body were likely to account for the above heterogeneity.

The current meta-analysis suggested fiber intake decreased the risk of SPs by 10% for the highest vs. lowest intakes. Dietary fiber affects colorectal cancer risk by increasing fecal volume, diluting fecal carcinogens in the colonic lumen, and shortening fecal transit time through the gut (54). Recent studies have shown that dietary fiber increases microbiota diversity in the gut (55) and gut microbiota is associated with CRC risk (56). A dose-response relationship meta-analysis found that each 10 g/d increase in the intake of cereal fiber was associated with a 9% lower risk of colorectal cancer (8), which supported our results.

The WHO has classified red meat as a class 2A carcinogen. A recent expert panel report also supported the role of red and processed meats in CRC risk (57). Processed red meat contains nitroso compounds that can cause DNA alkylation, which can lead to cellular carcinogenesis (58). Two meta-analyses performed by Zhao et al. manifested that compared with the lowest red meat intake, the highest red meat intake increased the risk of colonic adenomas and colorectal cancer (59, 60). In our study, red meat intake increased the risk of SP by 30%, which was consistent with the previous studies and added strong risk factor evidence.

The study provided a comprehensive analysis of associations between the above dietary factors and SP risk, which remain controversial today. Although most previous studies reported that calcium and folate intake could protect people from colorectal adenomas and cancer, the current meta-analyses found they had no such protective effect on SP.

This work was subjected to some shortcomings. First, as most studies were conducted based on U.S. populations, it is not appropriate to generalize our findings to African and Asian populations. Second, the results of folate and fiber intake may not be very stable according to sensitivity analyses. Besides, the included studies varied in many ways such as study design, adjustment for confounders, and different ascertainment methods for factor exposure, which might have an impact on the results. To minimize the effect of this limitation, we adopted random-effect models to pool the estimates. Subgroup analyses and meta-regression were also conducted to detect probable sources of heterogeneity, which enabled us to account for these differences.

## 5. Conclusions

The results of this meta-analysis indicate that higher dietary calcium, folate and fiber intake can reduce the risk of SP, and vitamin D intake may have the effect of preventing SPs, which needs to be determined by more evidence. Red meat intake is associated with an increased risk of SP. This evidence provides guidance for us to prevent SPs from a dietary perspective, such as moderately increasing fiber intake and reducing red meat intake. Further high-quality research is needed to clarify the role of vitamin D, folate, and calcium intake in SPs.

## Data availability statement

The original contributions presented in the study are included in the article/Supplementary material, further inquiries can be directed to the corresponding authors.

## Author contributions

XL, DX, and ZZ conceived and designed the meta-analysis. ZZ, XZ, XG, and NL searched the literature. ZZ, XZ, XG, NL, and SD extracted the data. ZZ analyzed the data, contributed analysis tools, and wrote the manuscript. XL revised the manuscript. All authors contributed to the article and approved the submitted version.

## Supplementary material

Supplementary figure 1 Risk of bias summary of RCTs.

Supplementary figure 2 Funnel plots with pseudo 95% confidence intervals.
