# Supplementary material for: Association between dietary factors and colorectal serrated polyps: a systematic review and meta-analysis
Source: Front Nutr. 2023 Jul 27;10:1187539. doi: 10.3389/fnut.2023.1187539 (PMC10413578; doi:10.3389/fnut.2023.1187539)
Supplement: Supplementary figure 1 — Risk of bias summary of RCTs. [file Data_Sheet_1.pdf]

Table S1. Quality assessment of included cross-sectional studies according to the Newcastle-Ottawa scale.

Cross-sectional studies	Selection (4)				Comparability (2)	Outcome (3)		Total
	Representativeness of the samples	Sample size	Non-respondents	Ascertainment of the exposure	Adjusted for covariates	Assessment of the outcome (2)	Statistical test	
Martinez, 1997	1	1	0	1	2	1	1	7
Adams, 2011	1	0	0	1	2	2	1	7
Crockett, 2015	1	0	1	1	2	2	1	8
Gurjar, 2019	1	0	0	1	2	1	1	6
Yoo, 2021	1	1	0	1	1	2	1	7
O'Sullivan, 2022	1	1	0	1	2	1	1	7

Case-control studies	Selection (4)				Comparability (2)	Exposure (3)			Total
	Is the case definition adequate (assessment of outcome)	Representativeness of the cases	Selection of controls (assessment of outcome)	Definition of controls (representativeness of the samples)	Adjusted for covariates	Ascertainment of exposure	Same method of ascertainment for cases and controls	Non-response rate	
Erhardt, 2002	1	0	0	1	2	1	1	1	7
Morimoto, 2002	1	1	0	1	2	1	1	1	8
Lieberman, 2003	1	0	0	1	2	1	1	1	7
Dai, 2007	1	0	0	1	2	0	1	1	6
Wallace, 2009	1	0	1	1	2	1	1	1	8
Burnett-Hartman, 2011	1	1	0	1	2	0	1	1	7
Fu, 2012	1	0	0	1	2	0	1	1	6
Shuai, 2016	1	0	1	1	2	1	1	0	7
Davenport, 2018	1	1	0	1	2	1	1	1	8
Ivancovsky-Wajcman, 2019	0	1	1	0	2	1	1	1	7

Mosley, 2020	0	1	1	1	2	1	1	1	8
Anthony, 2022	0	0	1	1	2	1	1	1	7

Cohort studies	Selection (4)				Comparability (2)	Outcome (3)			Total
	Representativeness of the exposed cohort	Selection of the non-exposed cohort	Ascertainment of exposure	Demonstration that outcome was not present at start of study	Adjusted for covariates	Ascertainment of outcome	Follow-up long enough for outcomes to occur	Adequacy of follow up of cohorts	
Kearney, 1995	1	1	1	1	2	1	1	0	8
Platz, 1997	0	1	1	1	2	0	1	1	7
He, 2018	1	1	1	1	2	1	1	1	9
Kim, 2021	1	1	1	1	2	1	1	1	9
Hang, 2022	1	1	1	1	2	1	1	1	9

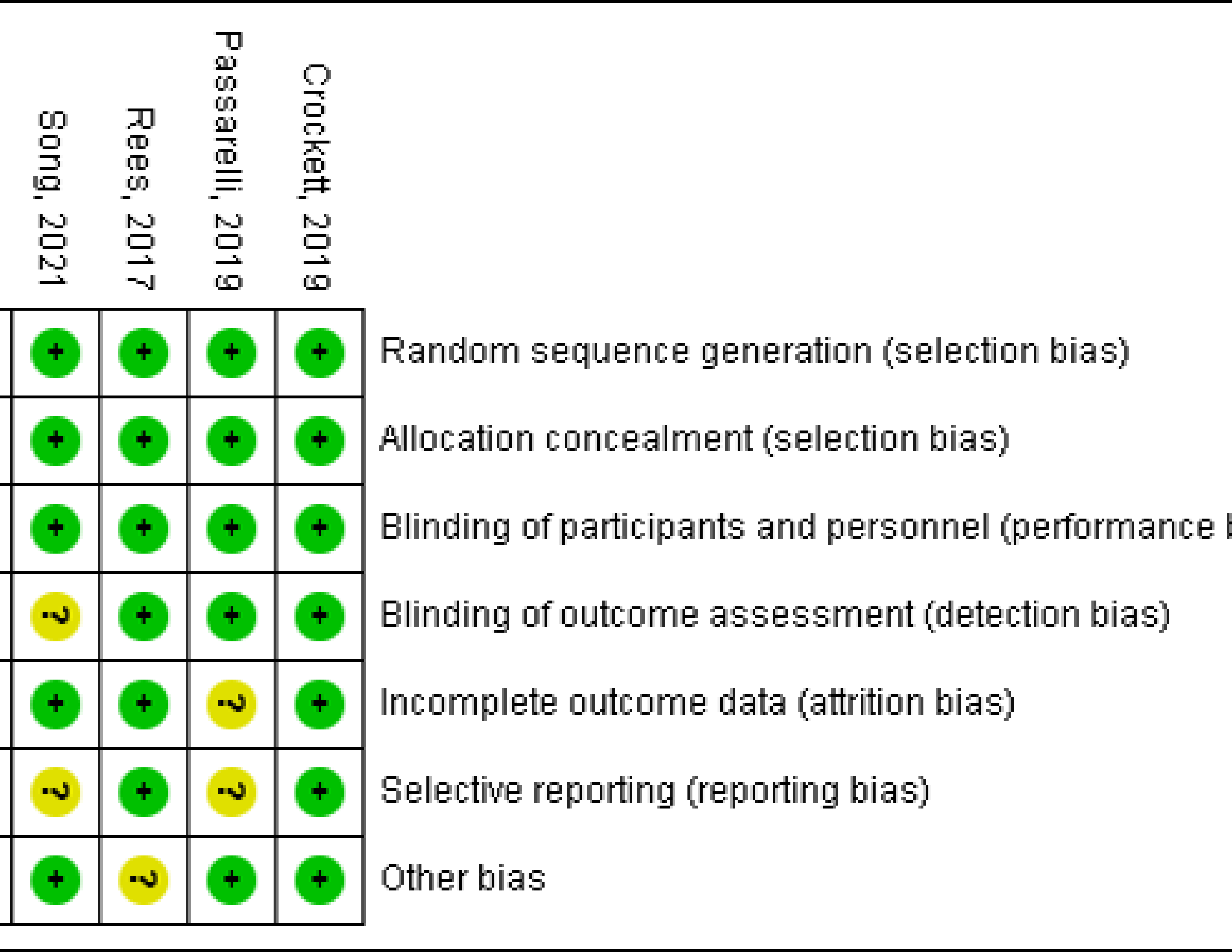
Song, 2021
Rees, 2017
Passarelli, 2019
Crockett, 2019
Random sequence generation (selection bias)
Allocation concealment (selection bias)
Blinding of participants and personnel (performance
Blinding of outcome assessment (detection bias)
Incomplete outcome data (attrition bias)
Selective reporting (reporting bias)
Other bias

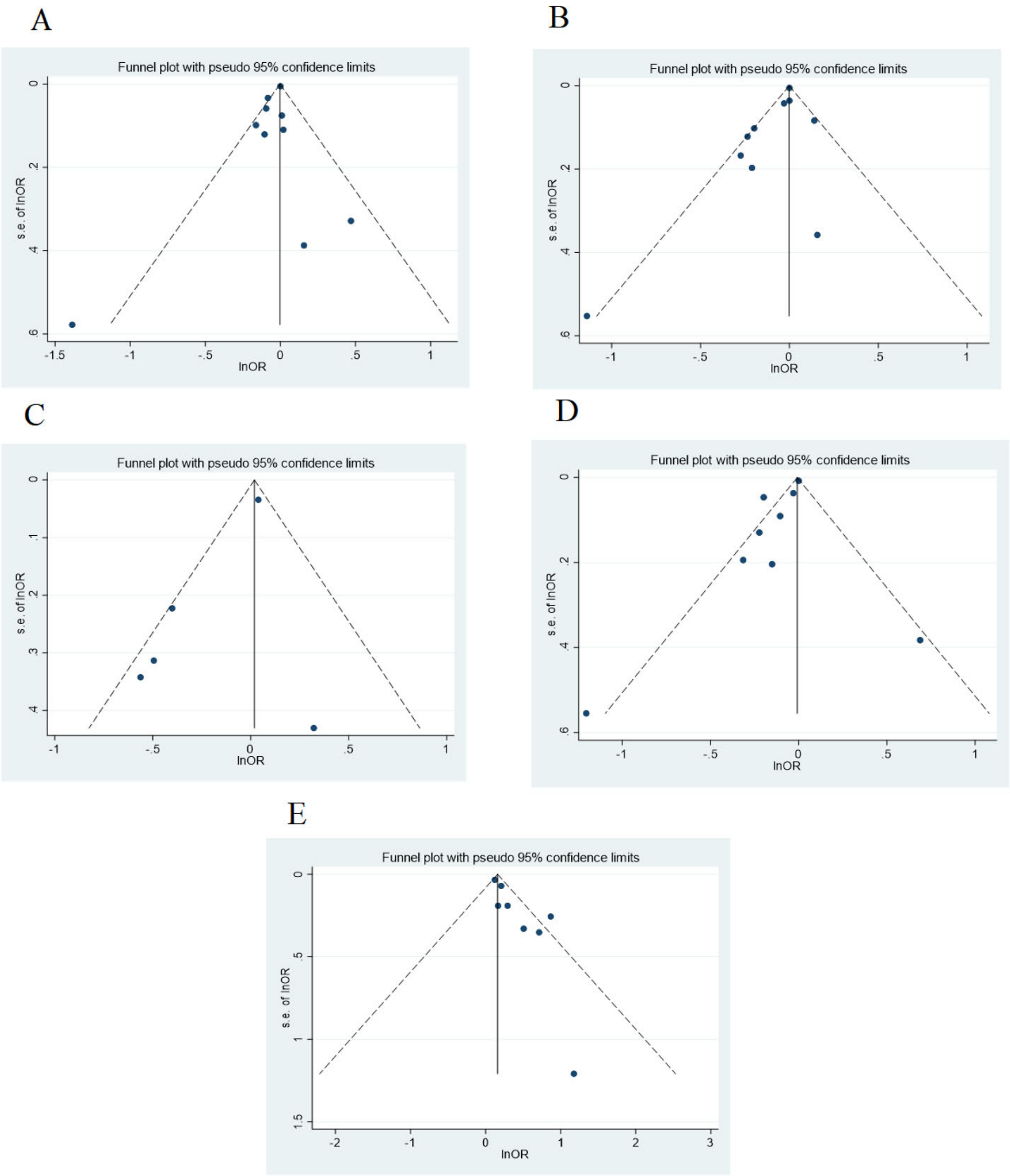
A
Funnel plot with pseudo 95% confidence limits
s.e. of lnOR
lnOR
B
Funnel plot with pseudo 95% confidence limits
s.e. of lnOR
lnOR
C
Funnel plot with pseudo 95% confidence limits
s.e. of lnOR
lnOR
D
Funnel plot with pseudo 95% confidence limits
s.e. of lnOR
lnOR
E
Funnel plot with pseudo 95% confidence limits
s.e. of lnOR
lnOR

Table S2. Results of Egger's publication bias test

Factors	t-value	*P*-value
Vitamin D	-1.54	0.162
Calcium	-1.93	0.090
Folate	-1.78	0.173
Fiber	-2.03	0.082
Red or processed meat	3.67	0.010

Pubmed:

(serrated OR hyperplastic) AND ((risk) AND ((("Adenomatous Polyps"[MeSH Terms] OR "Adenoma"[MeSH Terms] OR "Adenoma"[Text Word] OR "adenomas"[Text Word] OR "adenomatous"[Text Word] OR "Polyps"[MeSH Terms:noexp] OR "Intestinal Polyps"[MeSH Terms] OR "polyp"[Text Word] OR "Polyps"[Text Word]) AND ("intestine, large"[MeSH Terms] OR "Colorectal Neoplasms"[MeSH Terms:noexp] OR "Colonic Neoplasms"[MeSH Terms:noexp] OR "Rectal Neoplasms"[MeSH Terms:noexp] OR "colon"[Text Word] OR "rectum"[Text Word] OR "colonic"[Text Word] OR "rectal"[Text Word] OR "colorectal"[Text Word] OR "colo rectal"[Text Word]))))

Embase:

(serrated OR hyperplastic) AND ('large intestine tumor'/exp OR 'colon'/exp OR 'rectum'/exp OR colon:ab,ti OR rectum:ab,ti OR colonic:ab,ti OR rectal:ab,ti OR colorectal:ab,ti OR 'colo rectal':ab,ti) AND ((adenoma:ab,ti OR adeonmas:ab,ti OR adenomatous:ab,ti OR polyp*:ab,ti) OR 'colorectal adenoma'/exp OR 'intestine polyp'/exp OR 'adenoma'/exp) AND risk

Cochrane Library:

ID Search

#1 MeSH descriptor: [Polyps] explode all trees

#2 MeSH descriptor: [Adenoma] explode all trees

#3 (Adenomatous Polyps OR Adenoma OR adenomas OR adenomatous OR "Polyps OR Intestinal Polyps OR "polyp) (Word variations have been searched)

#4 risk

#5 #1 or #2 or #3

#6 ((colorectal OR colorectum OR colon* OR rectal OR rectum)) (Word variations have been searched)

#7 #4 and #5 and #6
